# Speech rehabilitation after total laryngectomy: long-term results with indwelling voice prosthesis Blom-Singer^®^

**DOI:** 10.1016/S1808-8694(15)31207-6

**Published:** 2015-10-20

**Authors:** Carlos Takahiro Chone, Ana L. Spina, Agricio N. Crespo, Flavio M. Gripp

**Affiliations:** 1 Ph.D. in Medicine, major in Otorhinolaryngology, Faculdade de Ciências Médicas, Universidade Estadual de Campinas, Assistant Physician, Discipline of Otorhinolaryngology and Head and Neck Surgery / Unicamp.; 2 Speech and Hearing Therapist, Master. Discipline of Otorhinolaryngology and Head and Neck Surgery, Unicamp.; 3 Ph.D., Joint Professor, Unicamp, Head of the Department of Ophthalmology-Otorhinolaryngology, Discipline of Otorhinolaryngology and Head and Neck Surgery, Unicamp.; 4 Assistant Physician, Otorhinolaryngology and Head and Neck Surgery Unicamp. Otorhinolaryngology and Head and Neck Surgery, Universidade Estadual de Campinas (Unicamp).

**Keywords:** câncer, laringe, reabilitação vocal, voz, prótese fonatória, cancer, larynx, voice rehabilitation, voice, voice prosthesis

## Abstract

To evaluate long-term use of indwelling Blom-Singer voice prosthesis (VP) for vocal rehabilitation of patients submitted to total laryngectomy (TL). We studied the influence of time of performance of tracheo-esophageal puncture (TEP), use of radiotherapy (XRT), patients’ age and length of follow-up, on the rate of success of use of VP. **Study Design**: clinical prospective. **Material and Method**: Seventy-one patients were submitted to TL and rehabilitated with indwelling VP. Both otolaryngologist and speech pathologist evaluated all patients for the vocal functional issues during the follow-up. The relative data on time of placement of VP, time of use of PF, use of XRT, age, length of follow-up and interval of duration of each VP were recorded during the follow-up. **Results**: There was 87% of patients with primary TEP and 13% with secondary. The follow-up varied from 12 to 87 months, with average of 38 months for primary and 51 months for secondary TEP. There were 59% of patients submitted to XRT. The general rate of success was of 94%. In primary TEP it was of 97% and in the secondary, it was 78% (p=0.07) and after two years, the success rate was of 96% in primary TEP and 75% in secondary TEP (p=0.07). The use of XRT and patient age did not influence the success of use of VP among primary and secondary TEP, independently of length of follow-up. **Conclusion**: Tendency to greater success rate in voice rehabilitation after TL with primary TEP was observed. Postoperative XRT and age did not influence success rate.

## INTRODUCTION

Since its introduction, the tracheo-esophageal puncture technique (TEP) and vocal prosthesis (VP) placement became the standard for vocal rehabilitation in patients submitted to total laryngectomy (TL) 1, 2, 3, 4, 5, 6, 7, 8, 9, 10, 11. Esophageal voice rehabilitation has a success rate of 24% to 26% 6, 12 and prosthesis with TEP has an increased success rate of 58% to 94% for immediate results of primary TEP and 61% to 64% for secondary TEP 6, 8, 10, 11, 13, 14, 15, 16, 17. Long-term results in the literature, with one-year follow up, ranged from 65% to 85% in primary and 69% to 83% in secondary approach 6, 10, 13, 14, 15, 17, 18, 19, 20, 21, 22. The main innovation in design and shape of prosthesis placement took place in the USA and Europe, with gradual and global improvement thanks to long-term use by patients ^4^.The most recent innovation was the introduction of indwelling VP to suppress the inconvenience and the problems associated with frequent exchanges of VP. The purpose of the present study was to assess the experience of indwelling voice prosthesis Blom-Singer^®^ in patients submitted to TL in a tertiary hospital, at the Discipline of Otorhinolaryngology, Head and Neck Surgery, Universidade Estadual de Campinas (Unicamp). We studied the influence of time of TEP performance (primary or secondary), use of postoperative radiotherapy (RTX postop), age of patients and follow-up over success rate of VP.

## MATERIAL AND METHODS

Seventy-one laryngectomized patients were submitted to vocal rehabilitation with VP between January 1995 and September 2001, following a minimum period of one year. Only patients with laryngeal squamous cell carcinomas cases submitted to total laryngectomy associated with neck dissection or postop radiotherapy were included in the study. Patients with malignant neoplasms of other sites that have also required simultaneous excision were excluded from the study.

Vocal rehabilitation of TL patients was exclusively made with indwelling VP Blom-Singer^®^ (Inhealth, Carpinteria, California).

Patients submitted to TL as of January 1995 were rehabilitated with VP through primary TEP. Patients submitted to TL before this date were submitted to secondary vocal rehabilitation with TEP.

Primary TEP is performed during TL and maintained opened with the placement of a nasogastric tube gage 14 for approximately 2 weeks. Once there is scaring of tracheostome and pharynx is confirmed, the VP is inserted. Secondary TEP is performed later after TL. The opening is maintained through a nasogastric tube for three days and then the VP is inserted. Criteria for indication of VP are well defined in the literature ^5^. Primary and secondary TEP technique has been described in previous articles. Patients submitted to secondary TEP were previously studied under swallowing videofluoroscopy with insufflation test to rule out stenosis of pharyngoesophageal segment (PES) or pharyngoesophageal spasm. Up to 1999, during TEP, patients were submitted to myotomy of pharyngeal constriction muscles as part of primary or secondary TEP technique. As of 1999, the procedure was no longer performed. Patients that presented PE spasm were submitted to treatment with Botulinium toxin injection. VP insertion requires the placement of a 22F dilator in the TEP area. The VP is inserted in an sheath of gelatin. It is placed on the posterior flange of VP to create a rounded extremity for insertion. It is placed into the insertion instrument. The dilator is removed and the prosthesis is placed in the TEP area. The gelatin cover is dissolved within approximately 30 seconds. The prosthesis is checked for correct position by turning it 360° without resistance. The posterior flange is opened against the esophageal wall with a fibronasopharyngolaryngoscope. After placement of the prosthesis, the patient was asked to express him/herself, to make sure that the vocal quality was functional. Instructions for cleaning techniques using pipettes and brushes were also given to the patients.

The duration of the procedure in relation to TL (primary or secondary), replacement or discontinuation of use were recorded. All patients were assessed jointly by Otorhinolaryngologist and speech and hearing therapist concerning vocal functional aspects. We assessed maximum phonation time, with three consecutive measurements and vocal perception analysis by both healthcare professionals. We considered as successful phonation prosthesis use when phonation was equal or greater than 8 seconds. Patients with suspicion of PES spasms at vocal perceptive analysis after TEP were submitted to swallowing videofluoroscopy with and without phonation and computed manometry of PES and treated with botulinium toxin injection. Treatment was considered successful when phonation time was improved to over 8 seconds. After 1999, no more myotomy of pharyngeal constrictor muscles was performed both in primary and secondary TEP. All patients were assessed concerning successful use of VP for one month, every three months up to one year, and then every 6 months after the first year of follow-up. Data concerning insertion time, duration of VP use, use of RTX postop, follow-up and duration of each VP were recorded during follow up of patients.

Patients whose margins were considered impaired or with presence of lymphatic emboli, perineural invasion, extralaryngeal extension, multiple metastases and extracapsular extension were referred to adjuvant treatment with RTXpostop.

The statistical analysis was performed with Fisher exact test. Results below 0.05 were considered significant.

The purpose of the study was to assess long-term use of indwelling Blom-Singer^®^ VP in patients submitted to TL in a tertiary hospital at the Discipline of Otorhinolaryngology and Head and Neck Surgery, Universidade Estadual de Campinas (Unicamp).

## RESULTS

In the group of patients submitted to primary TEP (62), 86% (53) presented follow up greater than 2 years. There were 38 (61%) patients submitted to RTX postop, out of which 33 (86%) presented follow up for more than 2 years. Twenty-four patients were not submitted to RTX postop and 20 (83%) were followed up for over 2 years. There was no difference in success rate between patients submitted to radiotherapy or not after two years of follow up. In the group with primary TEP, 32 (52%) were aged younger or equal to 60 years and 30 (48%) were aged over 60 years. Both age ranges presented a success rate of 97%. The success rate for the primary TEP group was 97%. Considering only patients whose follow up lasted more than 2 years, the success rate was 96%.

In the group of patients submitted to secondary TEP (9), 89% (8) presented follow up greater than 2 years. There were four patients (44%) submitted to RTX postop all of them followed up for more than 2 years. Five patients were not submitted to RTX postop, out of which 4 (80%) were followed up for more than two years. There were no statistically significant differences in success rate of VP use in patients with and without postop RTX after 2 years of follow up. Two patients were aged less than 60 years with success rate of 50% and seven patients aged over 60 years had success rate of VP use of 86%.

The success rate of secondary TEP was 78%. Considering only patients followed up for over 2 years, the success rate was 75%.

Among the two studied groups, primary and secondary TEP, there was no statistically significant difference concerning number of patients in follow up longer than two years (p=0.4), number of patients submitted to RTX postop (p=0.18), even considering the only patients with more than 2 years follow-up (p=0.24).

Success rate of VP use did not vary according to use of TEX postop by age in both groups (p>0.05). The time of installation of TEP, if during primary or secondary TL, presented tendency to better results when performed during the TL.

Four patients gave up the use of VP, two after secondary and two after primary TEP, owing to acquisition of esophageal voice during the use of VP.

## DISCUSSION

The rehabilitation of total laryngectomized patients with tracheoesophageal voice with VP, after primary or secondary TEP, proved to be a more reproducible method and with fewer complications to patients 1, 3, 5, 6, 7, 8, 9, 10, 11. Vocal rehabilitation with esophageal voice has success rate of 24 to 26% 6, 12, but with VP this rate is increased to 58% to 94% for immediate results with primary TEP and 61% to 64% for secondary TEP 6, 10, 13, 14, 15, 16, 17. Long-term results in the literature with one-year follow up ranged from 65% to 85% in primary and 69% to 83% in secondary TEP 6, 10, 13, 14, 15, 17, 18, 19, 20, 21, 22. This is one of the few studies that focused on long-term use of VP after primary and secondary TEP, especially with assessment of results after two years of follow up. The immediate success rate of the study was 100% in primary and secondary TEP. After two years of follow-up, the success rate was 96% for primary TEP and 75% for secondary TEP. There was higher success rate tendency in patients submitted to primary than to secondary TEP. Other authors have also demonstrated higher rates of success with primary rehabilitation than secondary one, but without statistical analysis 13, 15.

This tendency may be related with small number of patients in the secondary TEP group. Another possible explanation is the fact that early rehabilitation in this patients with primary TEP (on average 14 days after removal of tracheoesophageal tube and beginning of oral feeding), in this early postoperative stage, takes place when patients are more motivated for oral communication. In addition, it may be that in patients with secondary TEP, the central command and muscle plasticity are impaired because of absence for a long time of the need to have an airway protection mechanism, with absence of larynx. Moreover, these patients commonly develop other vocal adaptation mechanisms, such as pharyngeal phonation, which may hinder vocal rehabilitation with VP. There are also cases that had failed esophageal voice training, probably because of some PES problem. Further prospective randomized studies would be required in the group of total laryngectomized patients submitted to primary TEP and to secondary TEP in late postoperative period to assess whether the moment the TEP is performed actually influence the final result. However, as a result of the success with TEP rehabilitation, not to rehabilitate them at first and then submit patients to a second procedure under general anesthesia would not be ethically acceptable.

The use of postoperative radiotherapy did not show influence in the success of VP use in our study similarly to other studies 9, 14, 23, 24. Thus, the fact that total laryngectomized patients were previously submitted to radiotherapy or will be submitted to this treatment did not compromise phonation rehabilitation with VP.

Age was another factor that did not demonstrate influence in the success rate with the use of VP. Thus, advanced age of the patient is not a limiting factor for the use of VP, given that the indication criteria are respected ^5^ and depending on appropriate psychological, speech and medical guidance when the indication of TL is made and up to the day of the surgery concerning phonation rehabilitation with assessment of motivation and expectations ^5^.

Indwelling VP was designed to reduce the discomfort of making frequent changes of prosthesis for the patients. This need leads to inconvenience and potential problems (loss of PES segment, formation of granulation tissue and aspiration), especially in patients that present difficulties with manual dexterity, vision or motivation. The indwelling devices were not designed to improve speech, but when compared to short-life devices they are more comfortable because they require fewer changes, whereas the others require many changes, normally made by the patient, hindering compliance to treatment owing to more complex instructions ^4^. Thus, we used this type of indwelling prosthesis as the first option owing to its advantages. The habitual life of the prosthesis used in this study was 6.43 months and it exceeded the average of 7 weeks of the short-term prosthesis ^4^. The financial aspects of the indwelling device are also comparable to short-term devices ^4^. These prostheses are not completely free from maintenance. The use of systemic or topical nistatin for daily application with cleaning brush is necessary for most patients with any type of VP to prevent fungal colonization and leaks. However, some believe that it is more inconvenient to clean than to frequently change the VP, such as in short-term VP. The use of a length that allows tight adjustment of both flanges - tracheal and esophageal - of the prosthesis without tension (owing to formation of granulation tissue) or clearance (resulting liquid and saliva leak through the prosthesis) are important. The fact that frequent exchanges of VP are not required may increase the potential population of TL patients that can be rehabilitated by VP.

One of the causes of failure in vocal rehabilitation of patients submitted to TL with VP is spasm of PES 6, 25, 26, 27, 28, 29. This motor affection of PES is a reflex triggered by input of air into the esophagus and it prevents progression of airflow to the pharynx. Thus, there is no vibration of pharyngeal mucosa and phonation 9, 10, 27, 30, 31, 32, 33, 34, 35. The spasm may be observed in the videofluoroscopy during phonation with VP 12, 27, 28, 33, 35, 36, 37 and it is absent during swallowing, with relaxation of PES. In the constriction, there is no relaxation during swallowing. The treatment of this last case is dilation of PES 12, 27, 35. These are natural protection mechanisms against gastroesophageal reflux but in patients with TL they become an obstacle for phonation rehabilitation 10, 33, 34. There are three forms of treatment for PES affection: myotomy of medial and inferior pharyngeal constrictor muscles, neurectomy of pharyngeal plexus, and recently published, the technique for chemical denervation of LPS with botulinum toxin 1, 6, 7, 11, 28, 32, 33, 34, 36, 37, 38, 39, 40.

The use of botulinum toxin injection in LPS was initially used to treat the spasm after TEP with insertion of VP in 1995 by Blitzer et al. ^38^. There are authors who have demonstrated effects up to two years and three months or more, after initial application, without the need to reapply 7, 37. A possible explanation to this fact is that after initial application, the patients would get adapted to the new situation ^7^ or there would be denervation of pharyngeal constrictor muscle as a result of pre-synaptic blockage played by botulinium toxin. In the present study, six patients presented spasms confirmed by vocal perceptive analysis and swallowing videofluoroscopy. After application of 100 units of botulinium toxin under electromyographic control, they all presented improvement in phonation time, absent for over 8 seconds. Two patients after three years of follow up did not require application of botulinium toxin. Other four patients required one more application between 8 and 18 months after the first one.

In primary TEP, myotomy of medial and inferior constrictor muscles of the pharynx is one of the surgical times of the described surgical technique 1, 5. Its performance may be related with incidence of postoperative salivary fistula 8, 28, 34. As a result of this last occurrence, there may be consequent increase in time of hospitalization, hospital cost, delay in phonation rehabilitation, delay in introduction of oral feeding and up to beginning of postoperative radiotherapy of the patients. The real need of myotomy in TEP is controversial in the literature, and there are between 9% and 79% of patients submitted to LT^1^, ^8^, ^9^, ^10^, ^11^, ^25^, ^27^, ^30^, ^31^, ^33^, ^34^, ^35^. In secondary TEP, the conduction of myotomy is related to 10 to 20% of incidence of salivary fistulae ^5^, with similar consequences as previously described. The use of botulinium toxin to approach PES spasm rather than traditional myotomy allows the selection of only patients that really require treatment of PES and we can prevent unnecessary procedures in other patients, with consequent reduction of complications and surgical time. The injection of botulinium toxin is cheaper than myotomy of pharyngeal constrictor muscles ^7^. We should bear in mind that even though a myotomy of medial and inferior constrictors is performed, there may still be spasms because of muscle fiber approximation 1, 9, 11, 32, 33, when botulinium toxin can also be used. As of 1999, we have no longer performed myotomy in primary and secondary TEP and the patients that progressed with spasms, without improvement with speech therapy, are selected for treatment with botulinium toxin. Even with spasm, 75% of the cases progress with good voice six months after the procedures, without any other procedure involving the PES rather than speech therapy ^16^.

Patients that gave up use of VP in primary and secondary TEP amounted to 22% and 3%, respectively. These patients gave up use after 2 years of follow up and the reason was development of concomitant esophageal voice. After its acquisition, this was the method of choice for vocal rehabilitation, Maybe the use of tracheoesophageal voice has improved the skills these patients had to acquired esophageal voice. The patients that progressed with esophageal voice all preferred this method of vocal rehabilitation, probably owing to absence of need to have occlusion of tracheostome with the finger and the possibility of using both hands when talking and no need to change the prostheses, even if occasionally ^4^. Apparently, the preference for type of phonation rehabilitation in laryngectomized patients is esophageal voice ^41^, even though few patients can master it at first. Maybe VP has a role in its acquisition. The fact is when esophageal voice is acquired, the patients prefer this modality of vocal production, even if they had been already rehabilitated with VP ^41^, despite the fact that it has better vocal quality and acoustic performance 42, 43, 44, 45, 46. Further studies are required to understand the physiology of PES in total laryngectomized patients so that we can help them in acquiring the best form of vocal rehabilitation.

## CONCLUSION

The overall success rate of vocal rehabilitation in total laryngectomized patients with VP was 94% and it was better when performed in the first time (97%) than in the second time (78%). These results did not change after two years of follow up and did not suffer the impact of postoperative radiotherapy or age of patients.

The statistical analysis showed tendency to higher success rates in vocal rehabilitation in patients submitted to TL with primary TEP both overall and after two years of follow-up.
